# Emerging Role of LncRNA Regulation for NLRP3 Inflammasome in Diabetes Complications

**DOI:** 10.3389/fcell.2021.792401

**Published:** 2022-01-12

**Authors:** Xiaolin Lu, Qihong Tan, Jianyong Ma, Jing Zhang, Peng Yu

**Affiliations:** ^1^ The Second Clinical Medical College of Nanchang University, The Second Affiliated Hospital of Nanchang University, Nanchang, China; ^2^ Department of Pharmacology and Systems Physiology, University of Cincinnati College of Medicine, Cincinnati, OH, United States; ^3^ Department of Anesthesiology, The Second Affiliated Hospital of Nanchang University, Nanchang, China; ^4^ Department of Metabolism and Endocrinology, The Second Affiliated Hospital of Nanchang University, Nanchang, China

**Keywords:** NLRP3, inflammasomes, lncRNA, diabetes complications, antidiabetics

## Abstract

Diabetes is a widespread metabolic disease with various complications, including diabetic nephropathy, retinopathy, cardiomyopathy, and other cardiovascular or cerebrovascular diseases. As the prevalence of diabetes increases in all age groups worldwide, diabetes and its complications cause an emerging public health burden. NLRP3 inflammasome is a complex of several proteins that play a critical role in inflammatory response and various diseases, including diabetes and its complications. Accumulating evidences indicate that NLRP3 inflammasome contributes to the development of diabetes and diabetic complications and that NLRP3 inflammation inactivation is beneficial in treating these illnesses. Emerging evidences suggest the critical role of long non-coding RNAs (lncRNAs) in regulating NLRP3 inflammasome activity in various diseases. LncRNAs are non-coding RNAs exceeding 200 nucleotides in length. Its dysregulation has been linked to the development of diseases, including diabetes. Recently, growing evidences hint that regulating lncRNAs on NLRP3 inflammasome is critical in developing and progressing diabetes and diabetic complications. Here, we discuss the role of lncRNAs in regulating NLRP3 inflammasome as well as its participation in diabetes and diabetic complications, providing novel insights into developing future therapeutic approaches for diabetes.

## Introduction

Diabetes is a chronic metabolic disorder characterized by hyperglycaemia. Persistent hyperglycaemia and long-term metabolic disorders can damage a wide range of organs throughout the body, including diabetic nephropathy, retinopathy, cardiomyopathy, and many other complications (Forbes and Cooper, 2013; Reddy et al., 2015; Vujosevic et al., 2020). As the prevalence for all age groups increases worldwide, diabetes and its related complications not only impair physical and psychological properties of people but also impose a tremendous burden on society, both in economic and well-being terms (Cho et al., 2018). Therefore, a better understanding of the pathogenesis of diabetes and its complications is crucial for identifying therapeutic targets and developing effective medications.

The pathogenesis of diabetes and its complications is complex and encompasses a plethora of distinct pathways. Inflammation plays a vital role in diabetes and its complications, and the underlying mechanisms have been investigated for a prolonged time. An early clinical trial has indicated that high inflammation levels were strongly associated with type 2 diabetes (Bertoni et al., 2010). Chen G. and Goeddel D. V. depicted an authoritative tumor necrosis factor receptor-1 (TNF-R1)-mediated inflammatory signaling pathway that was implicated in the pathogenesis of diabetes (Chen and Goeddel, 2002). Obesity-associated diabetes causes an intensified crisis *via* numerous fat-derived molecules, such as IkappaB kinase, which seriously provoke inflammation (Lazar, 2005; Olefsky, 2009). In addition, previous research has proved that chronic inflammatory stimulation can cause a surge in plasma glucose levels by inhibiting the rate-limiting enzyme of bile acid biosynthesis, CYP7A1, which is linked to hepatic mevalonate pathway regulation (Okin and Medzhitov, 2016). Recently, several lncRNAs have been implicated in the inflammation associated with diabetes and its complications. Kato and others discovered that a megacluster of nearly 40 microRNAs (miRNAs) hosted by long non-coding RNA-megacluster (lnc-MGC) is coordinately upregulated to induce renal extracellular matrix accumulation and glomerular hypertrophy through cumulative effects in diabetic nephropathy (Kato et al., 2016). A study revealed that long non-coding RNA (lncRNA) and microRNA (miRNA) are correlated with inflammatory response, oxidative stress, apoptosis, hypertrophy, and fibrosis in diabetic cardiomyopathy, implying the development of new therapeutic and preventative strategies in diabetes complications (Jakubik et al., 2021).

**FIGURE 1:**
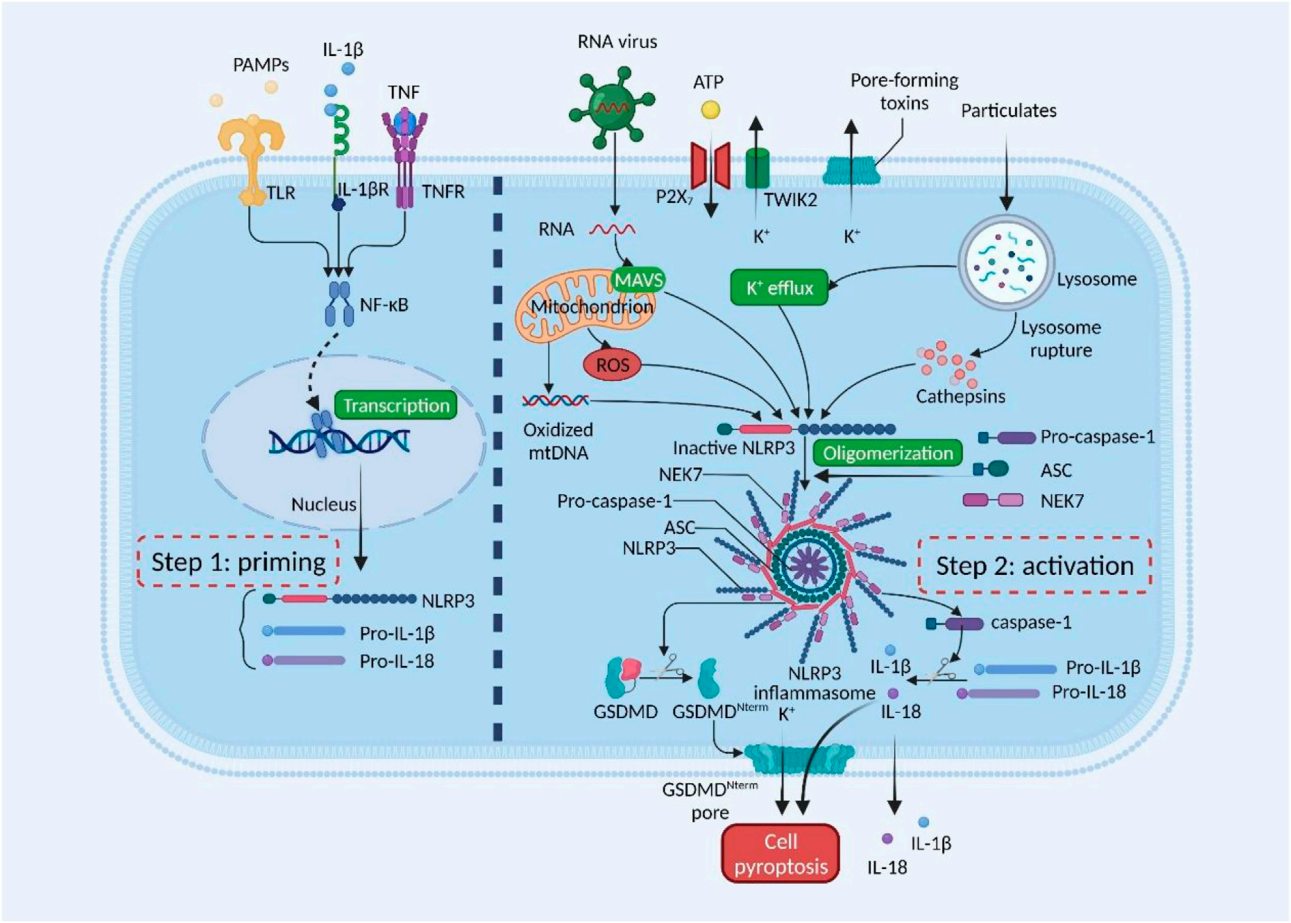
Mechanisms of NLRP3 inflammasome activation. NLRP3 inflammasome must be primed, followed by activation. The priming step is activated by pathogen-associated molecular patterns (PAMPs) or cytokines, leading to transcriptional upregulation of NLRP3, pro-IL-1β, and pro-IL-18. The activation step is induced by numerous PAMPs or damage-associated molecular patterns (DAMPs), such as particulates, pore-forming toxins, and ATP. RNA viruses activate NLRP3 through mitochondrial antiviral signaling protein (MAVS) on the mitochondrial outer membrane. NLRP3 inflammasome activates caspase-1, which in turn cleaves pro-IL-1β and pro-IL-18. Gasdermin D (GSDMD) is also cleaved and inserted into the membrane, forming pores and inducing pyroptosis. GSDMDNterm, GSDMD amino-terminal cell death domain; NEK7, NIMA-related kinase 7; NF-κB, nuclear factor-κB; P2X_7_, P2X purinoceptor 7; ROS, reactive oxygen species; TLR, Toll-like receptor; TNF, tumor necrosis factor; TNFR, tumor necrosis factor receptor; TWIK2, two-pore domain weak inwardly rectifying K+ channel 2. This figure was created with BioRender.com.

Currently, NLRP3 inflammasome activation is a prominent mechanism of inflammation response (Haneklaus et al., 2013; Swanson et al., 2019; Sharma and Kanneganti, 2021). NLRP3 inflammasomes are innate immune system protein complexes composed of NLRP3 (Figure 1); the adaptor protein apoptosis-associated speck-like protein (ASC), proinflammatory caspase, and caspase-1 (Swanson et al., 2019). NLRP3 is an intracellular sensor that detects a broad range of microbial motifs, endogenous danger signals, and environmental irritants. ASC is mainly distributed in the nucleus of human monocytes/macrophages. It quickly transfers to the cytoplasm under stress, connecting NLRP3 and pro-caspase-1. Caspase-1 is the effector protein of NLRP3 inflammasome, cleaved by the precursor molecule pro-caspase-1. Recently, NIMA-related kinase 7 (NEK7) is a serine-threonine kinase that appears to be a component specific to NLRP3 inflammasome (Shi H. et al., 2016; He et al., 2016; Schmid-Burgk et al., 2016). Upon inflammasome activation, NEK7 oligomerizes with NLRP3 into a complex essential for ASC speck formation and caspase-1 activation. Since inflammasome activation is an inflammatory process, it must be strictly regulated. With few exceptions, inflammasome activation is considered a two-step process (Guo et al., 2015; Swanson et al., 2019). First, it must be primed, and then it can be activated. The first step is to promote nuclear factor-κB (NF-κB) into the nucleus and upregulate the expression of NLRP3, caspase-1, and pro-IL-1β (Bauernfeind et al., 2009). This transcriptional upregulation can be induced through pattern recognition receptors (PRRs) such as Toll-like receptors (TLRs) recognizing various pathogen-associated molecular patterns (PAMPs) or damage-associated molecular patterns (DAMPs), or through cytokines such as TNF and IL-1β (Xing et al., 2017). The second step is activated by recognizing NLRP3 activators such as ATP, pore-forming toxins, viral RNA, or particulate matter. This cellular and molecular effect promotes the oligomerization of inflammasomes and leads to caspase-1-dependent release of pro-inflammatory cytokines IL-1β and IL-18, as well as gasdermin D (GSDMD)-mediated pyroptotic cell death (Rathinam and Fitzgerald, 2016; Swanson et al., 2019).

Increasing evidence demonstrates that NLRP3 inflammasome is implicated in developing diabetes and associated complications (Schroder et al., 2010; Wada and Makino, 2016; Tang and Yiu, 2020; Li et al., 2021). Diabetes and its complications activate NLRP3 inflammasome through hyperglycemia, hypercholesterolemia, and hyperuricemia, resulting in a rise of IL-1β and IL-18 levels and inducing inflammatory response (Schroder et al., 2010). The current study revealed that reducing NLRP3 inflammasome activation can prevent and reduce diabetic complications (Ashrafizadeh et al., 2021; Li et al., 2021). As an upstream regulator of NLRP3 inflammasome, lncRNA can exert control over diabetes and its complications (Li et al., 2017b). Understanding the mechanism by which lncRNA regulates NLRP3 inflammasome is significant to discover novel therapeutic targets for diabetes and its complications. This review summarizes the mechanism by which lncRNAs contribute to the development of diabetes and its complications by regulating NLRP3 inflammasome.

## The Mechanism of LncRNA Involved in NLRP3 Inflammasome Regulation

LncRNAs are linear non-coding RNAs having a length of more than 200 nucleotides (Hon et al., 2017). According to their relative protein-coding gene location in the genome, lncRNAs can be classified into five types (Table 1) (Ponting et al., 2009): (A) sense lncRNAs: their transcriptional direction is the same as that of neighboring protein-coding gene; (B) antisense lncRNAs: their transcriptional direction is opposite to that of neighboring protein-coding genes; (C) bidirectional lncRNAs: they can be simultaneously transcribed from the same and opposite direction with neighboring protein-coding genes; transcription occurs in the opposite two directions; (D) intronic lncRNAs: they can be transcribed from the intronic regions of genes; and (E) intergenic lncRNAs: they are derived from intergenic transcription of two genes. In addition, lncRNAs can be classified into four categories based on their biological functions (Table 1) (Guo et al., 2019): (A) signals lncRNAs: act as molecular signals or indicators of transcriptional activity; (B) decoy lncRNAs: bind to and sequester other regulatory RNAs or proteins; (C) guide lncRNAs: direct the localization of ribonucleoprotein complexes to specific targets; (D): scaffold lncRNAs: act as platforms for the assembly of relevant molecular elements (proteins and/or RNAs).

**TABLE 1:** Two classification kinds of lncRNAs.

Category	Feature
Classification based on genomic location	
Sense LncRNA	transcribed from the same direction
Antisense LncRNA	transcribed from the opposite direction
Bidirectional LncRNA	transcribed from the same and opposite direction
Intronic LncRNA	transcribed from intronic regions of genes
Intergenic LncRNA	transcribed from intergenic transcription of two genes
Classification based on function	
Signals LncRNA	act as molecular signal or indicator
Decoy LncRNA	bind to and sequester other regulatory RNAs or proteins
Guide LncRNA	direct the localization
Scaffold LncRNA	act as platform

Recent evidence indicates that lncRNAs play essential functions in many biological processes, such as X chromosome inactivation, dosage compensation, genomic imprinting, chromatin modification and remodeling, cellular proliferation, differentiation, and apoptosis (Kopp and Mendell, 2018; Nair et al., 2020). The mechanisms involved are mainly regulation at gene expression levels. In general, lncRNAs regulate gene expression through multiple pathways and molecular mechanisms at three levels: epigenetic, transcriptional, and post-transcriptional (Kopp and Mendell, 2018). On the one hand, lncRNAs can regulate gene expression at the epigenetic level (dosage compensating effects, chromatin modifications, and genomic imprinting). On the other hand, lncRNAs can be regulated at the transcriptional level by interfering with the transcription of messenger RNA (mRNA) or other non-coding RNAs, complexing with proteins, or acting through cis-acting elements (Gil and Ulitsky, 2020). Moreover, lncRNAs can be regulated at the post-transcriptional level by participating in mRNA degradation, regulating mRNA translation, and competitively binding miRNAs (Thomson and Dinger, 2016).

As known, lncRNAs not only regulate cell proliferation, differentiation, and metabolism but also participate in the pathological processes of various diseases, including cancer, diabetes, and neurodegenerative diseases (Boon et al., 2016; Leung and Natarajan, 2018; Feng et al., 2019; Guo et al., 2019). Diabetes-induced inflammation has been linked to the development of a variety of illnesses. As long-term hyperglycemia induces inflammatory response, vascular and target organs damage occurs (Nolan et al., 2011), ultimately increasing the incidence of tumors (Shikata et al., 2013; Singh et al., 2021), cardiovascular and cerebrovascular diseases (Kozakova et al., 2019; Eckel et al., 2021), and other infections (D'Elia et al., 1991; Knapp, 2013; Fang et al., 2021). NLRP3 inflammasome regulation by lncRNAs is a current research hotspot and has been widely documented in many diseases, such as inflammatory bowel diseases (Samoilă et al., 2020), Parkinson’s disease (Haque et al., 2020; Cao et al., 2021), and cancer (Farooqi et al., 2020; Tang et al., 2020). Studies indicate that lncRNAs and NLRP3 inflammasome are overexpressed in diabetes complications, implying that lncRNAs could cause inflammatory responses by activating NLRP3 inflammasome (Hu et al., 2019; Farooqi et al., 2020; Liu et al., 2020).

**FIGURE 2:**
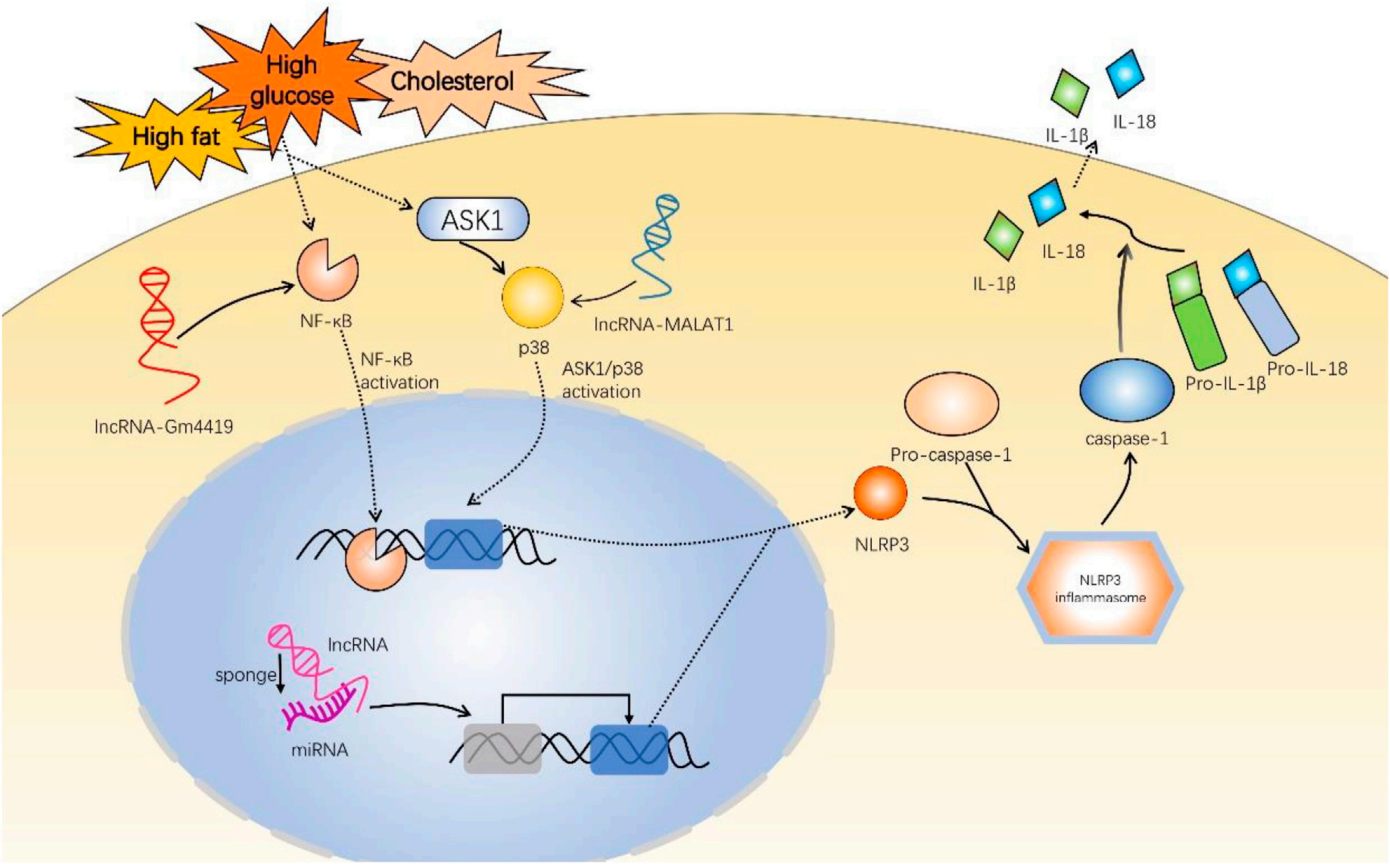
Mechanisms of long non-coding RNAs involved in inflammatory responses of diabetic complications *via* NLRP3 inflammasome. These experiments used high-glucose induction to establish diabetic mouse models. LncRNA-Gm4419 activates NF-κB pathway to upregulate NLRP3 expression in DN. LncRNA-MALAT1 activates ASK1/p38 pathway to upregulate NLRP3 in DR. In addition, most lncRNAs, such as ANRIL, Kcnq1ot1, NEAT1, HCP5, SNHG16, H19, and HCG18 promote or inhibit NLRP3 expression by sponging miRNA and regulating downstream target genes. NLRP3 and pro-caspase-1 are indispensable to NLRP3 inflammasome assembly. NLRP3 inflammasome activates pro-caspase-1 into caspase-1, promoting IL-1β and IL-18 generation. NF-κB, nuclear factor-κB; ASK1, apoptosis signal-regulating kinase 1; MALAT1, metastasis-associated lung adenocarcinoma transcript 1; lncRNA, long non-coding RNA; miRNA, microRNA; ANRIL, antisense noncoding RNA in the INK4 locus; Kcnq1ot1, Kcnq1 overlapping transcript 1; NEAT1, nuclear-enriched abundant transcript 1; HCP5, HLA complex P5; SNHG16, small nucleolar RNA host gene 16; HCG18, HLA complex group 18.

Notably, a vast number of studies have demonstrated that upregulation or downregulation of lncRNAs can inhibit NLRP3 inflammasome activation and reduce inflammatory response, hence improving diabetic complications (Xie et al., 2019; Liu et al., 2021). LncRNAs indirectly regulate NLRP3 inflammasome by acting as competing endogenous RNAs (ceRNAs) and sponging miRNAs (Figure 2). miRNAs could directly regulate downstream protein NLRP3 expression, ultimately affecting NLRP3/IL-1β pathway. Numerous investigations have demonstrated this lncRNA/miRNA-mediated NLRP3 inflammasome regulation mechanism in diabetic complications (Che et al., 2020c; Du et al., 2020; Liu et al., 2020; Xu et al., 2020; Wang and Zhao, 2021).

## Regulation of LncRNA on NLRP3 Inflammation in Diabetes and Its Complications

### Nephropathy

Diabetic nephropathy (DN) is a microvascular complication caused by diabetes-induced glomerular capillary damage. The main pathogenesis comprises glucose metabolism disorder, abnormal renal hemodynamics, extracellular matrix accumulation, abnormal expression of cytokines, genetic factors, and reactive oxygen species (ROS) formation (Wolf, 2004).

Numerous studies have demonstrated that inflammation plays a key role in DN pathogenesis, proving possible regulatory mechanisms (Matoba et al., 2019). NF-κB signal pathway is not only one of the principal inflammatory signal pathways in DN progression but also the signal pathway that governs DNA transcription *in vivo*. One recent study revealed that long intergenic noncoding RNA (lincRNA)-Gm4419 expression is elevated in mesangial cells (MCs) under a high glucose medium. Gm4419 can directly interact with P50, a functional subunit of NF-κB, activating NF-κB pathway. Meanwhile, P50 promotes transcription of NLRP3 inflammasome and inflammatory cytokines. These results indicate that Gm4419 may contribute to inflammation, fibrosis, and proliferation in MCs exposed to high glucose *via* NF-κB/NLRP3 inflammasome signaling pathway (Yi et al., 2017). NLRP3 inflammasome activation causes podocyte pyroptosis, proliferation of MCs, and renal tubular injury (Xiong et al., 2021). Interestingly, P50 can form a positive synergistic Gm4419 regulation in MCs (Yi et al., 2017).

Studies have indicated that lncRNAs can act as regulators by interacting with particular miRNAs in DN. Zhang C *et al.* found that lncRNAs promote podocyte pyroptosis by NLRP3 upregulation through interaction with microRNA (miR)-486a-3p. Additionally, podocytes were induced by sublytic complement C5b-9 (sC5b-9) *in vitro* (Zhang et al., 2021). This means that lncRNA/miRNA/NLRP3 signal pathway can be activated under specific conditions. Another experiment revealed that DN serum samples and high-glucose (HG)-treated MCs increased lncRNA-HLA complex P5 (HCP5) and high mobility group AT-hook 2 (HMGA2) expression and decreased miR-93-5p expression. Experiments have verified that lncRNA-HCP5 upregulates HMGA2 expression *via* miR-93-5p sponging. Additionally, HMGA2 can promote the release of inflammatory cytokines, such as TNF-α, IL-1β, and IL-6. Therefore, targeting lncRNA-HCP5/miR-93-5p/HMGA2 axis can inhibit hyperproliferation, fibrosis, and inflammation of HG-treated MCs (Wang X. et al., 2021). In addition, there are additional similar signaling pathways that have a similar function in DN (Li et al., 2017b; Liu et al., 2020; Zhan et al., 2020; Zhu et al., 2020). Specifically, long noncoding RNA-growth arrest-specific 5 (lncRNA-GAS5) expression was downregulated in HG-induced human renal tubular (HK-2) cells. Meanwhile, GAS5 overexpression could downregulate the expression of NLRP3, cleaved-caspase1, IL-1β, and GSDMD-N by directly targeting miR-452-5p (Xie et al., 2019). However, the specific inhibitory mechanism remains unclear. It may competitively inhibit NLRP3 expression by promoting the expression of specific downstream genes.

Thioredoxin-interacting protein (TXNIP) as a mediator of OS (oxidative stress) was implicated in activating NLRP3 inflammasome in DN progression (Samra et al., 2016). Both lncRNA-antisense noncoding RNA in the INK4 locus (ANRIL) and TXNIP expressions were significantly increased in DN kidney tissues and HG-treated HK-2 cells, whereas miR-497 was reduced. ANRIL has been proved to promote pyroptosis in DN, most likely *via* miR-497/TXNIP/NLRP3 pathway transmission (Wang and Zhao, 2021). Another research revealed that maternally expressed 3 (MEG3) knockdown resisted hyperoxia-induced lung cell pyroptosis by promoting miR-18a expression, whereas miR-18a inhibited TXNIP (Zou et al., 2020).

Overall, these outcomes reveal that lncRNA plays a critical part in DN pathogenesis (Table 2). In recent years, DN-specific processes, such as podocyte loss, glomerulosclerosis, and tubulointerstitial fibrosis, mediated by lncRNA through acting on NLRP3 inflammasome, have garnered considerable attention. However, the critical regulatory targets remain unknown. Further mechanism research is required to provide new ideas for DN treatment.

**TABLE 2:** LncRNAs regulating NLRP3 inflammasome in diabetes complications.

lncRNA	Expression	Target	Expression	Mechanism	Phenomenon	Diseases	Reference
Gm4419	↑	NF-κB	activate	activates NF-κB pathway	MCs pyroptosis	DN	Yi et al. (2017)
ANRIL	↑	miR-497	↓	↑TXNIP, ↑NLRP3	HK-2 pyroptosis	DN	Wang and Zhao, (2021)
Kcnq1ot1	↑	miR-486a-3p	↓	↑NLRP3	podocyte pyroptosis	DN	Zhang et al. (2021)
MALAT1	↑	miR-23c	↓	↑ELAVL1, ↑NLRP3	HK-2 pyroptosis	DN	Li et al. (2017a)
GAS5	↓	miR-452-5p	↓	↑NLRP3	HK-2 pyroptosis	DN	Xie et al. (2019)
Kcnq1ot1	↑	miR-506-3p	↓	↑NLRP3	HK-2 pyroptosis	DN	Zhu et al. (2020)
MALAT1	↑	miR-200c	↑	↑NRF2, ↑NLRP3	podocyte pyroptosis	DN	Zuo et al. (2021)
MALAT1	↑	miR-30c	↓	↑NLRP3	HK-2 pyroptosis	DN	Liu et al. (2020)
NEAT1	↑	miR-34c	↓	↑NLRP3	HK-2 pyroptosis	DN	Zhan et al. (2020)
HCP5	↑	miR-93-5p	↓	↑HMGA2 ↑TNF-α, IL-6, IL-1β	excessive proliferation, fibrosis and inflammation of MCs	DN	Wang et al. (2021a)
SNHG16	↑	miR-146a-5p	↓	↑IRAK1 activates NF-κB pathway	positively regulates proliferation, migration, and angiogenesis of hRMECs	DN	Cai et al. (2021)
SNHG16	↑	miR-7-5p	↓	↑IRS1 activates PI3K/AKT pathway		DN	Cai et al. (2021)
MALAT1	↑	p38	—	activates ASK1/p38 pathway, ↑NLRP3	positively regulates proliferation, migration, and angiogenesis of hRMECs	DR	Zou et al. (2021)
H19	↓	miR-19b	↑	↓SIRT1,↑TNF-α, IL-1β, IL-6	negatively regulates inflammatory responses of ARPE-19 hRMECs	DR	Luo et al. (2021)
GAS5	↓	miR-34b-3p	↑	↓AHR, ↑NLRP3	HL-1 cardiomyocytes pyroptosis	DCM	Xu et al. (2020)
MALAT1	↑	miR-141	↓	↑NLRP3	cardiac fibrosis	DCM	(Che et al., 2020c)
Kcnq1ot1	↑	miR-214-3p	↓	↑caspase-1, IL-1β	cardiac fibrosis	DCM	Yang et al. (2018)
GAS5	↑	miR-21-5p	↓	activates TLR4/NF- κB pathway	AC16 cardiomyocytes pyroptosis	DCM	Zhao et al. (2020)
HCG18	↑	miR-146a	↓	↑TRAF6,↑TNF-α, IL-1β, IL-6	M1 macrophage polarization	DPN	Ren et al. (2021)
PVT1	↑	miR-146a	↓	activates TGF-β/SMAD4 pathway, ↑TNF-α, IL-1, IL-6 and TGF-β1	promotes cartilage degradation	DOA	Wang et al. (2021b)

NF-κB, nuclear factor kappa light-chain enhancer of activated B cells; MCs, mesangial cells; ANRIL, antisense noncoding RNA, in the INK4 locus; miR, miRNA; TXNIP, thioredoxin-interacting protein; HK-2, human renal tubular cells; Kcnq1ot1, Kcnq1 overlapping transcript 1; MALAT1, metastasis-associated lung adenocarcinoma transcript 1; ELAVL1, ELAV-like RNA, binding protein 1; GAS5, growth arrest-specific 5; NRF2, nuclear factor erythroid-2-related factor 2; NEAT1, nuclear-enriched abundant transcript 1; HCP5, HLA, complex P5; HMGA2, high mobility group AT-hook 2; SNHG16, small nucleolar RNA, host gene 16; IRAK1, interleukin-1, receptor-associated kinase 1; IRS1, insulin receptor substrate 1; PI3K, phosphatidylinositol 3-kinase; hRMECs, human retinal microvascular endothelial cells; SIRT1, silence information regulator factor-related enzymes 1; ARPE-19, retinal pigment epithelial; AHR, aryl hydrocarbon receptor; TLR4, Toll-like receptor 4; HCG18, HLA, complex group 18; TRAF6, TNF, receptor associated factor 6; PVT1, plasmacytoma variant translocation 1; TGF-β1, transforming growth factor β1; SMAD4, mothers against decapentaplegic homolog 4; DN, diabetic nephropathy; DR, diabetic retinopathy; DCM, diabetic cardiomyopathy; DPN, diabetic peripheral neuropathy; DOA, diabetic osteoarthritis.

### Retinopathy

Diabetic retinopathy (DR) is a significant consequence of diabetes caused by diabetic microvascular disease. Approximately one-third of diabetic patients have DR (Wong et al., 2016). The pathophysiological processes of DR mainly include abnormal proliferation, migration, and neovascularization in the retina (Cai et al., 2021).

Although the mechanisms by which lncRNAs contribute to DR remain largely unclear, an increasing number of studies has demonstrated critical regulatory functions of various lncRNAs in microvascular dysfunction. For instance, Yan *et al.* has explored the mechanism of retinal microvascular dysfunction caused by diabetes and found that lncRNA-myocardial infarction-associated transcript (MIAT) expression increased in diabetic retinopathy. MIAT can act as a competing endogenous RNA, forming a feedback loop with vascular endothelial growth factor and miR-150-5p to regulate endothelial cell function, thereby contributing to pathological angiogenesis (Yan et al., 2015).

DR is a major cause of blindness in middle-aged and elderly patients. Visual function is a significant function of the body. If it can be protected early, it improves health-related quality of life in patients. Recently, one research has demonstrated that lncRNA small nucleolar RNA host gene 16 (SNHG16) upregulation in HG-stimulated human retinal microvascular endothelial cells (hRMECs) reduces proliferative DR-related abnormalities in cell proliferation, migration, and angiogenesis *via* regulating miR-146a-5p/interleukin-1 receptor-associated kinase 1 (IRAK1) and miR-7-5p/insulin receptor substrate 1 (IRS1) to activate NF-κB and phosphatidylinositol 3-kinase (PI3K)/AKT signaling pathways (Cai et al., 2021). It was also confirmed that SNHG16 exerts its function by isolating miR-146a-5p and miR-7-5p (Cai et al., 2021). Therefore, SNHG16 can guide individual therapy in DR. Another experiment revealed that in retinal pigment epithelial (ARPE-19) cells with HG conditions, lncRNA H19 and silence information regulator factor-related enzymes 1 (SIRT1) decreased while miR-19b increased. Besides, SIRT1 suppresses the expression of inflammatory cytokines, such as TNF-α, IL-1β, and IL-6 (Luo et al., 2021).

LncRNA metastasis-associated lung adenocarcinoma transcript 1 (MALAT1) knockdown prevents hyper-proliferation of retinal endothelial cells through p38 mitogen-activated protein kinase (MAPK) signaling (Liu et al., 2014). This year, another research has demonstrated that NLRP3 promoted tube formation and angiogenesis of retinal microvascular endothelial cells (Zou et al., 2021). Also, the findings indicated that NLRP3-mediated aberrant retinal angiogenesis in DR was regulated *via* apoptosis signal-regulating kinase 1 (ASK1)/p38 axis (Zou et al., 2021). Obviously, MALAT1 regulates diabetes-related retinal vessel function by activating ASK1/p38/NLRP3 signaling pathway.

### Cardiomyopathy

Diabetic cardiomyopathy (DCM) is a serious end-stage complication related to diabetes. Nowadays, it is recognized that DCM pathogenesis includes hyperglycemia, protein non-enzymatic glycosylation, oxidative stress, myocardial fibrosis, abnormal calcium ion transport, increased fatty acid oxidation, neuroendocrine function activation, etc. (Yilmaz et al., 2015).

Myocardial fibrosis is one of the main causes of DCM. One study revealed that lncRNA-MALAT1 is elevated in diabetic mice and cardiac fibroblasts (CFs) treated with high glucose. Melatonin has the function of reducing collagen production in CFs treated with high glucose. It suppresses lncRNA-MALAT1/miR-141-mediated inflammatory activation of NLRP3 inflammasome and transforming growth factor (TGF)-β1/Smads signaling to produce anti-myocardial fibrosis effects (Che et al., 2020b). Other researchers discovered that lncRNA-Kcnq1ot1 was highly upregulated in diabetic myocardial tissues and CFs cultured under high glucose. After silencing lncRNA-Kcnq1 overlapping transcript 1 (Kcnq1ot1), miR-214-3p can inhibit caspase-1 due to the competitive binding between Kcnq1ot1 and miR-214-3p turns into loosening. Additionally, its downstream inflammatory cytokine IL-1β reduces collagen deposition and myocardial fibrosis (Yang et al., 2018). Therefore, Kcnq1ot1/miR-214-3p/caspase-1 regulatory signaling pathway is critical for regulating DCM myocardial fibrosis.

Inflammation has a key role in DCM development and progression. A study in 2020 revealed that lncRNA-GAS5 expression was upregulated in AC16 cardiomyocytes induced by high glucose. Additional studies indicated that lncRNA-GAS5 could competitively bind to miR-21-5p. Because miR-21-5p targets Toll-like receptor 4 (TLR4), silencing GAS5 can partially inhibit miR-21-5p-mediated TLR4/NF-κB signaling pathway, hence reducing inflammatory response triggered by high glucose (Zhao et al., 2020). However, another study concluded the opposite result. Xu, Y. *et al.* induced cardiac muscle cell line (HL-1) cardiomyocytes by high glucose and found that GAS5 was severely downregulated in DCM mice. Further experiments revealed that GAS5 overexpression could inhibit NLRP3 activation by regulating miR-34b-3p/aryl hydrocarbon receptor (AHR) signaling pathway, thereby reducing cardiomyocytes pyroptosis (Xu et al., 2020). The different results may be due to differences between cell lines or because multiple regulatory pathways coexist in the cell. As DCM is a complication of diabetes, the principal Frontier of its research is self-evident. To summarize, these results imply that lncRNA might be an underlying therapeutic target for DCM by alleviating NLRP3 inflammasome activation, fibrosis, and apoptosis.

### Peripheral Neuropathy

Diabetic peripheral neuropathy (DPN) is a ubiquitous complication of diabetes. Redox-sensitive transcription factors such as NF-кB play a critical role in triggering the cascade of cytokine and chemokine production, including proinflammatory cytokines IL-1β, IL-6, TNF-α, etc. (Zhou and Zhou, 2014). These are key inflammatory factors downstream of NLRP3 inflammasome and are involved in inflammatory response of DPN, which can not only enhance inflammation and immune response but also promote activation of various downstream cell oxidative stress pathways. Wang, C. *et al.* employed whole-transcriptome sequencing technology to systematically analyze the differential expression of lncRNAs, mRNAs, and miRNAs in Schwann cells (SCs) of DPN and control rats and constructed lncRNA–miRNA–mRNA competing endogenous RNA (ceRNA) network of SCs. This network has identified the inhibited relationship of lncRNA, miRNA, and mRNA and underlined that they function as key mediators in the pathophysiological process of SCs in DPN (Wang et al., 2020). This ceRNA regulatory network has a particular clinical application value in DPN. Another recent array study demonstrated that four lncRNAs, namely XR_353891, XR_600244, XR_595664, and XR_598132, can regulate inflammation signaling pathways by competitively binding with miR-146a-5p in DPN rats using qRT-PCR (Feng et al., 2020). LncRNA HLA complex group 18 (HCG18) promotes the polarization of M1 macrophages and DPN progression by regulating miR-146a/TNF receptor-associated factor 6 (TRAF6) axis. Additionally, in DPN model, inflammatory factors, such as TNF-α, IL-1β, and IL-6, are upregulated (Ren et al., 2021).

### Other Diabetes Complications

Diabetic foot is a serious complication of diabetes, mainly caused by DPN, peripheral vascular diseases, or infection. As known, lncRNAs usually act as sponges for microRNAs to exert their regulatory effects. However, some ceRNAs in diabetic feet require additional investigation. For instance, inhibiting miR-217 can upregulate hypoxia-inducible factor-1 (HIF-1α)/vascular endothelial growth factor (VEGF) pathway to promote angiogenesis in diabetic foot ulcer rats and effectively improve inflammatory response by decreasing inflammatory factors (IL-1β, TNF-α, and IL-6) (Lin et al., 2019). Furthermore, emodin has been demonstrated to protect diabetic foot through miR-9 upregulation and modulation of PI3K/AKT and NF-κB signaling pathways in neuron-like PC-12 cells (Fan et al., 2018). Consequently, it is imperative to further investigate the interaction and contrast relationship between lncRNA and miRNA to better cure diabetic foot.

The incidence of diabetic osteoarthritis (OA) increases, making it critical to identify an exact therapy. One research has verified that lncRNA plasmacytoma variant translocation 1 (PVT1) promoted cartilage degradation in diabetic OA mice by downregulating miR-146a and activating TGF-β/mothers against decapentaplegic homolog 4 (SMAD4) signaling pathway. It has been demonstrated that silencing PVT1 decreases the expression of proinflammatory mediators such as TNF-α, IL-1, IL-6, and TGF-β1, hence alleviating joint inflammation (Wang Y.-Z. et al., 2021). Interestingly, another experiment confirmed that PVT1 silencing could act as a sponge for miR-149 to combat metabolic imbalance to catabolism and inflammation after IL-1β exposure (Zhao et al., 2018). It provided a new direction for diabetic OA treatment.

### Potential Drugs Targeting LncRNA-Regulated NLRP3 Inflammasome in Diabetes and Its Complications

Melatonin can be produced by the pineal gland and is also present in various plants. It possesses pharmacological activities, including antioxidant, anti-inflammatory, liver protection, heart protection, and neuroprotection properties. In recent years, the effect of treating cancer and diabetes has been demonstrated (Ashrafizadeh et al., 2021). The clinical use of melatonin is controversial (Garaulet et al., 2020), but there are now some basic studies on its mechanism for different diabetes complications. For instance, melatonin has been demonstrated to have a protective effect of alleviating cardiac fibrosis on DCM by inhibiting lncRNA MALAT1. miR-141-5p, which acts as a sponge of MALAT1, inhibits the expression of NLRP3 inflammasome and TGF-β1/Smads signaling (Table 3) (Che et al., 2020c). TGF-β1 can initiate cardiac fibrosis *via* regulating extracellular matrix proteins in cardiac fibroblasts through activating Smads-mediated signal pathways in diabetic mice (Chen et al., 2015). In another work, melatonin can inhibit OS and inflammation by enhancing the activity of long non-coding RNA MEG3/miR-204/Sirt1 axis in experimental DR rats (Tu et al., 2020). Sirt1 can deacetylate the target gene forkhead box o1 (Foxo1) and the subunit p65 of NF-κB, leading to downregulation of inflammatory factors (Tu et al., 2021). Therefore, melatonin may be implemented as a potential agent for treating diabetic neuropathy (Che et al., 2020a).

**TABLE 3:** Mechanism of drugs treating diabetic complications.

Drugs	Diseases	Mechanism	Reference
Melatonin	Diabetic Cardiomyopathy	inhibits lncRNA MALAT1/miR-141-mediated NLRP3 inflammasome and TGF-β1/Smads signaling	(Che et al., 2020c)
Melatonin	Diabetic Retinopathy	inhibits NLRP3 inflammasome by upregulating MEG3/miR-204/Sirt1 axis	Tu et al. (2020)
Metformin	Diabetic Periodontitis	inhibits lncRNA NEK7 to improve NLRP3 inflammasome-mediated pyroptosis	Zhou et al. (2020)
Atorvastatin	Diabetic Neuropathy	inhibits NLRP3 expression by regulating MALAT1/miR-200c/NRF2 axis	Zuo et al. (2021)
Sinapic acid	Diabetic Atherosclerosis	inhibits lncRNA-MALAT1 to downregulate NLRP3 expression	Han et al. (2018)

MALAT1, metastasis-associated lung adenocarcinoma transcript 1; miR, miRNA; TGF-β1, transforming growth factor β1; MEG3, maternally expressed 3; NEK7, NIMA-related kinases 7; NRF2, nuclear factor erythroid-2-related factor 2.

Metformin has become a ‘foundation therapy’ for treating diabetes due to its excellent efficacy and safety. As a first-line treatment option for diabetes, metformin effectively controls the amount of glycogen when used alone or in combination with other drugs, such as sulfonylurea, thiazolidinedione, DPP-4 inhibitor, SGLT2 inhibitor, and GLP-1 receptor agonist or insulin (Sanchez-Rangel and Inzucchi, 2017). Recently, metformin has been confirmed to regulate lncRNA-mediated NLRP3 inflammasome in diabetes complications. Diabetic periodontitis is caused by diabetes leading to excessive inflammatory response of periodontal microbiome, and it subsequently increases insulin resistance (Lalla and Papapanou, 2011). NIMA-related kinases 7 (NEK7) is an essential mediator of NLRP3 activation downstream of potassium efflux (He et al., 2016). One research has revealed that metformin suppresses NEK7 expression in diabetic periodontitis to improve NLPP3 inflammasome-mediated pyroptosis (Zhou et al., 2020). Therefore, with additional research into the mechanism of action, metformin has a good clinical treatment prospect in diabetes and its complications, which warrants much attention.

Atorvastatin (AT) has lipid-decreasing, anticoagulant, antioxidative and anti-inflammatory functions (Shi M.-M. et al., 2016). Clinically, AT is often universally applied to treat lipid abnormalities and related angiopathies. Nuclear factor erythroid-2-related factor 2 (NRF2) plays a tremendous role in regulating OS and is lowly expressed under HG environments (Uruno et al., 2015). For example, one study has indicated that NRF2 hyperactivation can induce nephrogenic diabetes insipidus in early renal tube development (Suzuki et al., 2017). LncRNA MALAT1 is thought to be intimately linked to pyroptosis in diabetes complications (Li et al., 2017a). Interestingly, MALAT1 can stabilize and activate NRF2 in human umbilical vein endothelial cells under H_2_O_2_ disposed (Zeng et al., 2018). miR-200c overexpression can promote OS in endothelial cells and interact with MALAT1 structurally (Li et al., 2016; Carlomosti et al., 2017). In addition, Zuo Y *et al.* demonstrated that AT suppresses caspase-1, GSDMD, and NLRP3 expressions by regulating MALAT1/miR-200c/NRF2 activation to prevent podocyte pyrolysis and OS induced by high glucose (Zuo et al., 2021). It opens a new door to AT-induced therapy for diabetes complications.

Sinapic acid, a small naturally occurring hydroxycinnamic acid, contains 3,5-dimethoxyl and 4-hydroxyl substitutions in the phenyl ring of cinnamic acid. Sinapic acid is well known to show antioxidant, anti-inflammatory, anticancer, antiglycemic, and neuroprotective activities (Chen, 2016). Numerous regulation mechanisms for sinapic acid in diabetes and its complications have been revealed (Zych et al., 2019; Alaofi, 2020; Altındağ et al., 2021). Diabetic atherosclerosis is caused by chronic inflammation, dyslipidemia, and vascular endothelial injury under high blood sugar levels and is also an important cause of death and disability in diabetic patients (Giacco and Brownlee, 2010). It was stated that sinapic acid could alleviate inflammatory responses *via* inhibiting NLRP3 inflammasome activation (Lee et al., 2021). Recently, a study revealed that low-dose sinapic acid inhibits lncRNA-MALAT1 to downregulate NLRP3 expression, thereby alleviating macrophage pyroptosis in diabetic atherosclerosis rats (Han et al., 2018). These outcomes have revealed that sinapic acid has potential therapeutic value for diabetes complications.

In short, NLRP3 inflammasome is required for the inflammatory signaling pathway. IL-1β, IL-18, caspase-1, caspase-11, and NF-κB are important inflammatory factors in the inflammatory signaling pathway, whose expression indicates the role of NLRP3 inflammasome (Al Mamun et al., 2021). Simultaneously, the function of NLRP3 inflammasome provides a vital theoretical basis for using lncRNA as a therapeutic target to treat diabetes complications.

## Conclusions and Future Perspectives

This review discussed the role and potential regulatory mechanism of lncRNAs on NLRP3 inflammasome, presented recent progress on the functional role of lncRNA-linked NLRP3 inflammasome regulation for developing and progressing various diabetes complications, and illustrated potential medications that might be useful in preventing and treating diabetes and its complications. This opens up potential new avenues to treat diabetes and its complications by targeting lncRNA-linked NLRP3 inflammasome.

At present, with lncRNA as the target, research on the role of drugs to interfere with diabetic complications is in its infancy. LncRNA intervention has been demonstrated to affect initiation and activation of NLRP3 inflammasome, as well as the expression of its downstream genes, consequently inhibiting the occurrence and development of diabetic complications. However, the specific mechanisms still require in-depth studies.

As a key role in developing and progressing diabetes complications, NLRP3 inflammasome brings new research directions for preventing and treating diabetes complications in the future. The treatment of NLRP3 inflammasome inhibition by targeting lncRNA of specific inflammatory signaling pathways may become a novel strategy for delaying the progression of diabetic complications in the future. Given the complexity of diabetes pathogenesis and its complications, lncRNA regulation on NLRP3 inflammasome has been investigated.

Additional research is required to elucidate the role and mechanism of lncRNA-linked NLRP3-inflammasome regulation in diabetes complications and other inflammatory diseases.
